# Sudden Deafness as a Presenting Symptom of Acoustic Neuroma: Case Report

**DOI:** 10.1016/S1808-8694(15)30134-8

**Published:** 2015-10-19

**Authors:** Sérgio Marquez Nascentes, Eduardo Augusto de Oliveira Henrique Paulo, Eduardo Carvalho de Andrade, Ana Lúcia da Silva, Trissia Maria Farah Vassoler, Adriana Bernardini Antunes Scanavini

**Affiliations:** a Specialist in otorhinolaryngology - ABORLCCF; b Specialist in otorhinolaryngology - ABORLCCF; c Specialist in otorhinolaryngology - ABORLCCF; d Specialist in otorhinolaryngology - ABORLCCF; e Specialist in otorhinolaryngology - ABORLCCF; f Specialist in otorhinolaryngology -ABORLCCF. M.S. in Otorhinolaryngology - FMRP-USP

**Keywords:** acoustic neuroma, acoustic schwannoma, sudden deafness, tinnitus

## Abstract

Vestibular schwannoma, also known as acoustic neurinoma, is the most frequent tumor of the cerebellopontine angle, and represents 9% of all intracranial tumors. **Case Report:** The authors report a case of sudden deafness with unilateral tinnitus. The patients responded to therapy with Prednisone and Pentoxifylline after the diagnosis of acoustic neurinoma by imaging exams. **Discussion:** Sudden deafness can be described as an intense and abrupt sensorineural loss. Usually it is higher than 30 dB at three or more frequencies and develops in less than three days. **Conclusion:** Investigation of the etiology of sudden deafness is extremely important to establish the adequate strategy for the case.

## INTRODUCTION

Sudden hearing loss may be a case of abrupt and intense sensorineural hearing loss. It is usually above 30 dB, involving three or more contiguous frequencies, and sets-in in less than three days^1^. Patients usually know precisely when it happened, it is normally unilateral, and it may be permanent or not.

The causes of sudden hearing loss frequently raise doubts and controversies as to etiology, evolution and treatment. Virus is the predominant etiology, and it may have vascular, autoimmune, thyroid glad, LUES, labyrinthine fistulas, ototoxicity, trauma, neuropathies and schwannomas as possible causes^2^.

We know that tumors of the cerebellopontine angle, such as vestibular schwannomas may cause sudden hearing loss.

Vestibular schwannomas, also known as acoustic neuromas, are the most frequent type of tumor of the pontine angle, corresponding to approximately 9% of all intracranial tumors. It is a benign tumor that springs from the Schwann cells, more frequently in the upper branch of the vestibular nerve. It grows slowly towards the pontine angle, pushing against the VIII cranial nerve and enlarging the internal acoustic meatus^3^.

Most of the times, initial symptoms are neurotological: progressive unilateral sensorineural hearing loss, tinnitus and vertigo. When it reaches bigger sizes, it may not only affect the facial and the trigeminal nerves (facial paralysis, tear reduction, hypogeusia in the anterior 2/3 of the tongue, and no stapedial reflex with conductive hearing loss), but also present central neurological manifestations. The labyrinthine alteration does not happen in a crisis fashion - differentiating it from Meniere’s disease^4^.

The sudden unilateral hearing loss caused by the tumor is the major symptom, happening in up to 26% of the acoustic nerve tumors. A spasm or occlusion of the labyrinthine artery as the tumor pushes against it may be the reason for hearing loss.

In diagnosis, besides a detailed history and physical exam, it is necessary to run a complete audiologic evaluation with vestibular tests, to assess the trigeminal nerve, and to do a gadolinium contrasted MRI^5^.

Treatment of choice is surgical removal, and the access approach will depend on its location and extension.

The authors report a case of recurrent sudden hearing loss and tinnitus caused by a vestibular schwannoma.

## CASE REPORT

W. A. O., 38 years old, caucasian, married, repair shop manager, born in Bauru/ SP. Went to an otorhinolaryngologist for the first time in January of 2004, complaining of sudden left ear hearing loss without any triggering factors, lasting for two days already. He also complained of continuous high-pitch tinnitus in the same ear. He had no vertigo or neurological alterations.

He did not report prior pathologies (High blood pressure, diabetes, otitis, head injuries, upper airway infections) and use of medications.

He was normal under physical examination, with intact and shiny bilateral tympanic membranes, mild nasal septum deviation to the right side, tonsils grade I, no palpable lymph nodes.

His audiogram showed severe sensorineural hearing loss after 3000Hz, with SRI of 92% and SRT of 15 dB in his left ear; his right ear was within normal limits.

Based on this we suspected of sudden hearing loss and ordered a blood work up. The patient was started on Prednisone 60 mg/day and Pentoxifylline 800mg/day.

**Figure 1:**
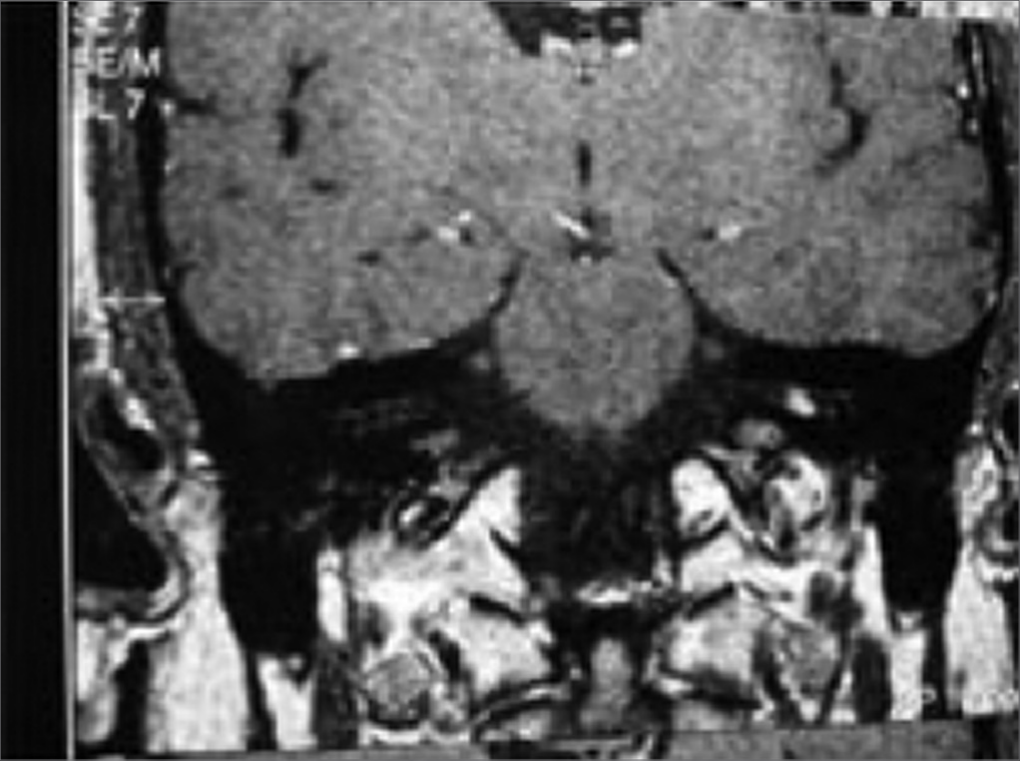
MRI with Vestibular Schwannoma, coronal view.

**Figure 2:**
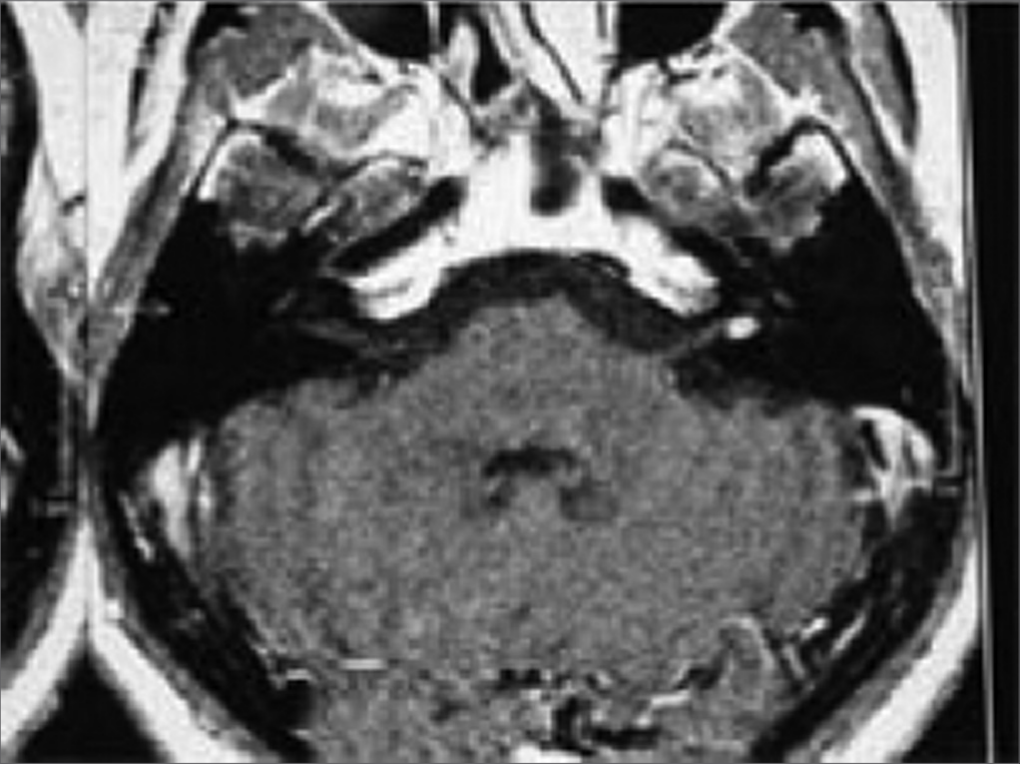
MRI with Vestibular Schwannoma, axial view.

**Chart 1:**
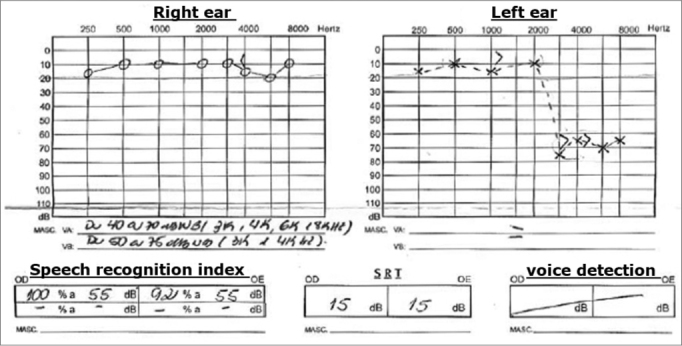
Audiogram 1.

**Chart 2:**
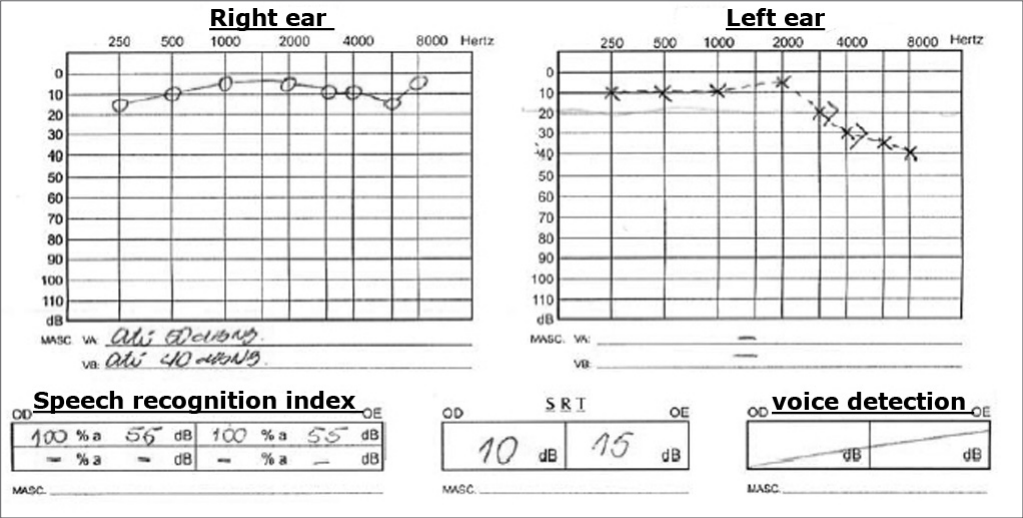
Audiogram 2.

**Chart 3:**
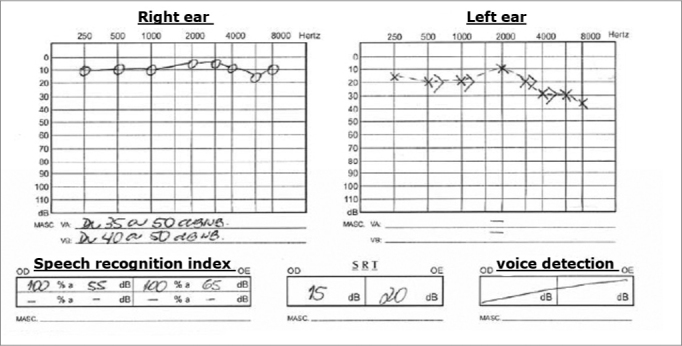
Audiogram 3.

After one week the patient returned saying he had a partial improvement in both hearing and tinnitus.

Lab exams: CBC normal; fasting glucose 103 mg/dL, total cholesterol 175 mg/dL, triglycerides 481 mg/dL, VDRL negative.

A new audiogram did not show alterations in his right ear and showed a light sensorineural hearing loss after 3000 Hz in his left ear, SRI 100%.

The patient was educated as to a better diet and medicated with Gingko biloba 240mg/day. We ordered an MRI and BERA.

The patient returned five months later, with a new manifestation of hearing loss and a worsening in his tinnitus in the left ear, with four days of evolution and he did not have vertigo.

Audiogram showed severe sensorineural hearing loss in the high frequencies in this left ear - similar to the first one. He was once again medicated with prednisone 60 mg/day and Pentoxifylline 800 mg/day, and reported a little improvement after he started with the medication.

Another MRI was ordered, this time showing an expansive solid mass, measuring 5mm in diameter, inside the internal acoustic meatus (vestibular schwannoma).

The patient was referred to a neurosurgeon, who decided to observe him clinically.

## DISCUSSION

Sudden hearing loss may be described as an abrupt and intense sensorineural hearing loss. It is usually above 30 dB, in three or more contiguous frequencies and takes less than three days to develop. Patients know exactly when it started, it is usually unilateral and may be permanent or not.

The causes of sudden hearing loss frequently bring doubts and controversies as to its etiology, evolution and treatment. It is known that tumors of the pontine angle, such as vestibular schwannomas may cause sudden hearing loss. Symptoms onset and duration are not strictly related to tumor size^6^.

Hearing loss in cases of vestibular schwannoma is usually unilateral and progressive, however it may happen suddenly and involve hearing discrimination more than auditory acuity.

Sudden hearing loss usually occurs thanks to an occlusion of the labyrinthine artery. It is yet unknown whether this sudden occlusion is caused by extrinsic compression alone or if there is any intravascular involvement (thrombosis), or a problem on the vessel wall (wall thickening), or some neuroendocrine factor affecting the artery (vasoconstriction)^3^.

We presented a case in which sudden hearing loss and tinnitus were the only symptoms the patient had, for whom we initially ordered audiometric exams, followed by laboratory exams and the patient was put on Prednisone and Pentoxifilin^7^, ^8^. The treatment was clinically successful until the etiological diagnosis, obtained by means of an MRI scan. With our case we discuss the efficacy and indication of clinical treatment in cases of sudden hearing loss without known etiology, and the importance of the MRI, even in patients who improve with the initial treatment^9^.

## FINAL COMMENTS

It is highly important to investigate the etiology in cases of sudden hearing loss, in order to better manage and treat such a patient. The otorhinolaryngologist must have in mind all the possible etiologies of sudden hearing loss and the complementary tests available for diagnosis.
